# Supplementary material for: Insights from a workplace SARS-CoV-2 specimen collection program, with genomes placed into global sequence phylogeny
Source: PLoS One. 2023 Apr 28;18(4):e0285042. doi: 10.1371/journal.pone.0285042 (PMC10146508; doi:10.1371/journal.pone.0285042)
Supplement: Attachment — Submitted filename: 20230320_rebuttal_letter3.docx [file pone.0285042.s001.docx]

902 Battelle Boulevard

P.O. Box 999, MSIN P7-50

Richland, WA 99352

(509) 375-2934

Kristin.omberg@pnnl.gov

**www.pnnl.gov**

March 20, 2023

Dr. Babatunde Olanrewaju Motayo

Academic Editor
PLOS ONE

Dear Dr. Motayo,

Thank you for your recent feedback regarding our manuscript. We have revised the manuscript in response to your feedback as well as the comments from Dr. Oderinde. These changes have been tracked in our revised manuscript in accordance with PLOS ONE requirements, and we respond to each individual comment below.

Our raw data have been deposited in the NCBI Sequence Read Archive (BioProject accession PRJNA891756) We look forward to acceptance of this manuscript, and hope it proves useful for the readers of PLOS ONE.

Sincerely,

Kristin M. Omberg, PhD

1. Please ensure that your manuscript meets PLOS ONE's style requirements, including those for file naming. The PLOS ONE style templates can be found at <https://journals.plos.org/plosone/s/file?id=wjVg/PLOSOne_formatting_sample_main_body.pdf> and <https://journals.plos.org/plosone/s/file?id=ba62/PLOSOne_formatting_sample_title_authors_affiliations.pdf>

**Response**: We identified author affiliations out of order and have re-numbered affiliations to meet requirements. We have also adjusted punctuation and spacing on the title page to better reflect that in the template.

2. Please make sure that all information entered in the 'Ethics Statement' section regarding ethics approval and informed participant consent is also included in the Methods section of the manuscript.

**Response:** We have added this information to the beginning of the methods section.

3. Please provide additional details regarding participant consent. In the ethics statement in the Methods and online submission information, please ensure that you have specified what type you obtained (for instance, written or verbal, and if verbal, how it was documented and witnessed). If your study included minors, state whether you obtained consent from parents or guardians. If the need for consent was waived by the ethics committee, please include this information.

**Response:** We have added language clarifying that written informed consent was obtained from all subjects.

4. We note that you have stated that you will provide repository information for your data at acceptance. Should your manuscript be accepted for publication, we will hold it until you provide the relevant accession numbers or DOIs necessary to access your data. If you wish to make changes to your Data Availability statement, please describe these changes in your cover letter and we will update your Data Availability statement to reflect the information you provide.

**Response:** We have provided repository information in our cover letter. Our raw data have been deposited in the NCBI Sequence Read Archive under accession PRJNA891756. We have also included this information in the relevant section of our methods.

5. We note that you have included the phrase “data not shown” in your manuscript. Unfortunately, this does not meet our data sharing requirements. PLOS does not permit references to inaccessible data. We require that authors provide all relevant data within the paper, Supporting Information files, or in an acceptable, public repository. Please add a citation to support this phrase or upload the data that corresponds with these findings to a stable repository (such as Figshare or Dryad) and provide and URLs, DOIs, or accession numbers that may be used to access these data. Or, if the data are not a core part of the research being presented in your study, we ask that you remove the phrase that refers to these data.

**Response:** As these comparisons were not part of the core research of this study, we have removed references to these data in the manuscript.

6. Please review your reference list to ensure that it is complete and correct. If you have cited papers that have been retracted, please include the rationale for doing so in the manuscript text, or remove these references and replace them with relevant current references. Any changes to the reference list should be mentioned in the rebuttal letter that accompanies your revised manuscript. If you need to cite a retracted article, indicate the article’s retracted status in the References list and also include a citation and full reference for the retraction notice.

**Response:** We have checked the list of references and to our knowledge all references are current and correct, and have not been retracted.

Review Comments to the Author

Reviewer #1: The research has added to knowledge about SARS-CoV-2 infection and in concept of susceptibility generally.

1. The Abstract should be more defined by mentioning the pool by percentage where the 36 positives emerged.

**Response:** We have edited the abstract to include this information.

2. The positive samples tested and analyzed should have a prevalence or percentage from the population tested and or the samples are selected a pool of positives which should be mentioned based on Ct values ranges or symptoms manifested by subjects.

**Response:** We have included information regarding percent positive of total screened patients. Given the voluntary nature of testing for this study, it would be reasonable to assume that patients experienced symptoms consistent with flu-like illness. However, because these symptoms were not recorded for this dataset, we have not made references to clinical symptoms in the manuscript.

3. There is no need to indent the paragraph, spacing adequately is ideal.

**Response:** We have left paragraphs indented in the body of the manuscript in accordance with journal style guidelines.

Line 71- Viral RNA extracted (NOT Isolated).
Line 34 – Statistical (NOT Statical)

**Response:** We are unsure exactly to which lines the reviewer refers but have made changes to isolated/extracted at line 97 and have corrected the statical/statistical typo at line 111.

4. The statistical analysis was properly done but some of the figure representations are not properly presented especially figure 5 should re-presented using another template to represent the Age Cohort / Gender clearly.
The figures should be labelled underneath of each of the figures with a written legend.

**Response:** We thank the reviewer for the comment regarding figure presentation, but respectfully disagree that the representation of age/gender cohort is not presented clearly in this figure. This figure was designed such that multiple comparisons between age/gender cohort can be made visually by the reader from a single figure – other representations of the data such as scatter plots do not reflect the distribution of and mean Ct values for each assay while representations such as violin plots do not allow for quick visual comparison between cohort pairs.

Each cohort of age and gender is clearly notated by distinct colors, chosen from a palate specifically designed to be distinguishable by readers with color-blindness. Additionally, the figure legend explicitly describes horizontal lines as mean Ct values for each qPCR assay, outliers, etc. However, we have adjusted the text in this section to define cohorts being tested more clearly as well as their relationships to each other. Finally, the figures are not labeled as submitted because PLOS ONE style guidelines are specific that figure legends should appear in the body of the manuscript after the paragraph in which each figure is referenced.
